# Supplementary material for: The Mixing Ratio and Filling-Amount Affect the Tissue Browning and Antioxidant Properties of Fresh-Cut Baby Leaf Lettuce (Lactuca sativa L.) and Rocket (Eruca sativa Mill.) Grown in Floating Growing Systems
Source: Foods. 2022 Nov 4;11(21):3515. doi: 10.3390/foods11213515 (PMC9654129; doi:10.3390/foods11213515)
Supplement: Supplementary file 1 [file foods-11-03515-s001.zip › foods-1984465-supplementary.pdf]

Supplementary Table S1. *Pearson correlation analysis of the selected variables.*

Variables		AC	TP	AA	PAL
AC	Pearson Correlation	1	0.770**	0.535**	0.608**
	Sig. (2-tailed)		*0.000*	*0.001*	*0.000*
	N	36	36	36	36
TP	Pearson Correlation		1	0.549**	0.596**
	Sig. (2-tailed)			*0.001*	*0.000*
	N		36	36	36
AA	Pearson Correlation			1	0.690**
	Sig. (2-tailed)				*0.000*
	N			36	36
PAL	Pearson Correlation				1
	Sig. (2-tailed)				
	N				36

N = number of replicates; **Correlation is significant at the 0.01 level (2-tailed); *Correlation is significant at the 0.05 level (2-tailed).
